# Acute effects of sports with different metabolic loads on serum irisin, BDNF, and NRF2 levels and cognitive performance in sedentary young adults: a comparative analysis of basketball and tennis protocols

**DOI:** 10.3389/fpubh.2026.1911856

**Published:** 2026-08-03

**Authors:** Cemre Can Akkaya, Canan Ceylan

**Affiliations:** 1 Faculty of Sport Sciences, Department of Coaching Education, Istanbul Yeni Yuzyil University, Istanbul, Türkiye; 2 Faculty of Sport Sciences, Department of Physical Education and Sports Teaching, Istanbul Sabahattin Zaim University, Istanbul, Türkiye

**Keywords:** acute exercise, basketball, BDNF, cognitive performance, irisin, muscle-brain axis, biomarkers, motor control

## Abstract

**Background and objective:**

Physical exercise triggers coordinated neurobiological responses. This study compared the acute effects of high-intensity intermittent (basketball) versus moderate-intensity continuous (tennis) exercise on serum irisin, BDNF, and NRF2 levels, and on working memory and inhibitory control in sedentary young adults (18–25 years).

**Methods:**

Sixty sedentary male participants (*n* = 20 per group) completed basketball, tennis, or passive control protocols. Serum biomarkers were quantified by ELISA pre- and post-exercise. Cognitive performance was assessed using the Digit Span and Stroop Color-Word Tests. Non-parametric analyses (Wilcoxon, Kruskal–Wallis, Dunn–Bonferroni) were applied, with a linear mixed-effects model used as a supplementary robustness check for serum irisin.

**Results:**

Both exercise groups showed significant within-group increases in all biomarkers and cognitive measures (*p* < 0.001), with no changes in controls (*p* > 0.05). Basketball produced greater irisin (+41.9% vs. +30.5%), BDNF (+63.9% vs. +29.9%), NRF2 (+73.5% vs. +47.1%), and cognitive improvements than tennis (all *p* < 0.01).

**Conclusion:**

Acute high-intensity intermittent exercise elicits significantly stronger neurotrophic, antioxidant, and cognitive responses than moderate-intensity continuous exercise in sedentary young adults, consistent with intensity-dependent activation of the muscle-brain axis; sport-specific motor and cognitive-load differences may also contribute and cannot be fully disentangled from metabolic intensity in the present design.

## 1 Introduction

The global surge in sedentary behavior has emerged as one of the principal modifiable risk factors for non-communicable diseases, neurodegeneration, and cognitive decline. According to the World Health Organization (1), approximately 1.4 billion adults worldwide are insufficiently physically active, underscoring the urgency of evidence-based exercise prescription strategies that target molecular and neurological mechanisms. Among the most compelling molecular targets of exercise-induced adaptation are the myokine irisin, the neurotrophin brain-derived neurotrophic factor (BDNF), and the cytoprotective transcription factor nuclear factor erythroid 2-related factor 2 (NRF2) three biomarkers that collectively orchestrate metabolic reprogramming, neuroplasticity, and cellular redox homeostasis (2–4).

Irisin, encoded by the FNDC5 gene and cleaved proteolytically from its membrane precursor fibronectin type III domain-containing protein 5, is a contraction-induced myokine primarily regulated via the PGC-1α/FNDC5 signaling axis (3, 5). Upon skeletal muscle contraction, PGC-1α coactivator expression is upregulated in proportion to exercise intensity, leading to FNDC5 transcription and subsequent irisin secretion into peripheral circulation. Critically, irisin traverses the blood–brain barrier and upregulates hippocampal BDNF expression, forming the molecular basis of the “muscle-brain axis” (6). This crosstalk positions irisin as a metabolic mediator connecting peripheral exercise stimuli to central neuroplastic adaptation.

BDNF, the most abundantly expressed neurotrophin in the mammalian brain (2), binds its high-affinity receptor tropomyosin receptor kinase B (TrkB) to activate downstream PI3K/Akt and MAPK/ERK pathways, promoting neuronal survival, dendritic arborization, synaptic potentiation, and hippocampal neurogenesis (7). Acute bouts of exercise transiently elevate circulating BDNF levels in proportion to exercise intensity and duration (8, 9), with peak concentrations typically occurring within minutes of exercise cessation. This transient elevation has been mechanistically linked to improved working memory, executive function, and learning consolidation (10, 11).

NRF2, the master regulator of antioxidant defense, is activated by reactive oxygen species (ROS) generated during muscular contraction. Elevated ROS oxidize cysteine residues on KEAP1, the principal NRF2 repressor, releasing NRF2 for nuclear translocation and transcriptional activation of phase II detoxifying enzymes including heme oxygenase-1 (HO-1), NAD(P)H quinone oxidoreductase 1 (NQO1), and superoxide dismutase [SOD; (4)]. Emerging evidence suggests bidirectional NRF2-BDNF signaling, wherein NRF2-mediated antioxidant upregulation protects BDNF from oxidative degradation, amplifying neuroprotective capacity following exercise (12). Despite this mechanistic convergence, studies simultaneously quantifying irisin, BDNF, and NRF2 in response to sport-specific acute exercise remain scarce.

Basketball and tennis represent two of the most globally practiced sports with markedly distinct metabolic profiles. Basketball is characterized by repeated maximal accelerations, decelerations, jumps, and directional changes, imposing intense demands on the phosphagen and anaerobic glycolytic systems interspersed with aerobic recovery an intermittent high-intensity exercise (HIIE) paradigm that maximally recruits type II myofibers and induces substantial metabolic acidosis (13). Tennis, by contrast, involves longer rally sequences and sub-maximal court displacements, relying predominantly on aerobic oxidative metabolism with moderate anaerobic contributions at a relative intensity of 60–75% HRmax (14). These differing metabolic stimuli may differentially activate PGC-1α/FNDC5/irisin, BDNF, and NRF2 cascades, thereby producing sport-specific magnitudes of neurocognitive adaptation.

Beyond metabolic intensity, acute exercise has also been linked to domain-specific cognitive effects. Verbal working memory and inhibitory control the two cognitive domains assessed in the present study appear particularly responsive to acute exercise, plausibly reflecting catecholaminergic and neurotrophic modulation of hippocampal-prefrontal circuitry (10, 11, 15, 16). However, the relative contribution of exercise intensity versus the task-specific perceptual-cognitive demands of a given sport (e.g., basketball’s open-skill, reactive decision-making versus tennis’s more predictable, closed-skill drill structure) to these cognitive gains remains insufficiently understood.

However, a critical gap remains in the literature: studies comparing the acute molecular and cognitive effects of these two sports modalities in genuinely sedentary young adults are absent. Most prior work has been conducted on trained athletes, limiting translation to the growing population of sedentary individuals for whom exercise prescription is most urgently needed. Additionally, methodological limitations including insufficient sample sizes, failure to account for non-normal biomarker distributions, and absence of validated cognitive outcome measures have constrained interpretation of previous findings.

This study was therefore designed with three primary objectives: (1) to quantify within-group pre-to-post acute exercise changes in serum irisin, BDNF, and NRF2 in basketball players, tennis players, and a sedentary control group; (2) to compare between-group difference scores across all biomarkers and cognitive measures; and (3) to establish an intensity-dependent hierarchy of exercise-induced neurotrophic and antioxidant responses in a sedentary young adult population. We hypothesized that acute basketball exercise, by virtue of its greater metabolic intensity, type II fiber recruitment, and open-skill cognitive demands, would produce significantly higher irisin, BDNF, and NRF2 elevations and greater cognitive improvements compared to tennis, with the control group remaining unchanged.

## 2 Materials and methods

### 2.1 Study design and ethical approval

This study employed a pre-test/post-test, parallel-groups (non-crossover) experimental design. All procedures were conducted in accordance with the ethical standards of the 2013 revision of the Declaration of Helsinki. Written informed consent was obtained from all participants prior to enrollment. The study was conducted between January and April 2026 at a foundation university in Istanbul, Türkiye.

### 2.2 Sample size determination and G*power analysis

*A priori* statistical power analysis was performed using G*Power 3.1.9.7 software (17). A large effect size (Cohen’s *f* = 0.45) was assumed for between-group biomarker comparisons; this assumption was based on effect sizes reported in prior acute irisin and BDNF exercise-intervention literature (3, 9) and represents an *a priori* estimate rather than a value derived from pilot data in the present sample. With a significance threshold of *α* = 0.05 and a desired statistical power of 1 − *β* = 0.85 for a Kruskal–Wallis *H* test with three independent groups, the minimum total sample size required was *N* = 54 (*n* = 18 per group). To account for potential dropout, measurement failure, and eligibility exclusions during the experimental phase, the target sample was inflated by approximately 11%, yielding a final allocation of *n* = 20 per group (*N* = 60). This sample size also provided ≥90% power for within-group Wilcoxon Signed-Rank tests under equivalent effect size assumptions.

### 2.3 Participants

Sixty sedentary male participants aged 18–25 years were enrolled across three groups: Basketball (BB; *n* = 20), Tennis (TEN; *n* = 20), and Sedentary Control (CON; *n* = 20). Participants were recruited via campus-wide advertisements at a local university in Istanbul. Sedentary status was operationally defined as engagement in less than 150 min per week of moderate-intensity physical activity over the preceding 6 months, as assessed by the International Physical Activity Questionnaire–Short Form [IPAQ-SF; (18)]. All participants classified as ‘low physical activity’ on the IPAQ-SF were eligible; those classified as ‘moderate’ or ‘high’ were excluded. None of the participants were competitive or recreational athletes, and none reported any structured exercise program participation during the preceding 6 months. Although participants were screened to exclude athletes, basketball- and tennis-specific motor familiarity accumulated through informal, non-competitive play (e.g., recreational school physical-education exposure) was not formally quantified with a validated instrument; this is addressed further in Limitations, as residual between-sport differences in movement efficiency cannot be fully excluded.

General inclusion criteria were: (i) male sex; (ii) age 18–25 years; (iii) non-smoker; (iv) body mass index (BMI) 18.5–29.9 kg/m^2^; (v) no chronic cardiovascular, metabolic, neurological, or musculoskeletal pathology; (vi) no pharmacological agents known to affect BDNF, oxidative stress markers, or cognitive function; (vii) normal color vision (confirmed by Ishihara plate test); (viii) no vigorous physical activity in the 48 h preceding testing. Exclusion criteria additionally included: dietary supplement use within the preceding 4 weeks, history of head trauma or concussion, a self-reported psychiatric diagnosis, and co-enrollment in another research study. Psychiatric-diagnosis exclusion was based on participant self-report during the intake interview rather than a standardized structured clinical instrument (e.g., the MINI or SCID); this is noted explicitly here, and as a limitation in Section 4, to avoid any ambiguity about the nature of this screening.

Participants were instructed to abstain from caffeine and alcohol for 24 h and to arrive at the laboratory in a fasted state (≥10 h) on the day of testing.

Participants were allocated to the Basketball, Tennis, and Control groups prior to any baseline assessment. A fully prospective, formally documented randomization protocol (e.g., a computer-generated allocation sequence with concealed envelopes) and a complete CONSORT-style participant flow record (numbers assessed for eligibility, excluded with reasons, and analyzed) were not retained in a form that can be reconstructed for this revision. We acknowledge the absence of a documented formal randomization and allocation-concealment procedure as a methodological limitation of the present study (see Section 4), and we have adopted prospective trial registration with full CONSORT flow documentation as standard practice for our ongoing and future studies.

Participant characteristics are presented in Table 1. Groups were comparable in age, body mass, height, and BMI (all *p* > 0.05 on Kruskal–Wallis *H* test), confirming successful demographic homogeneity. All control participants fulfilled IPAQ-SF ‘low physical activity’ criteria.

**Table 1:** Participant characteristics (median; IQR: 25th–75th percentile).

Variable	Basketball (*n* = 20)	Tennis (*n* = 20)	Control (*n* = 20)	*H*	*p*
Age (years)	21.5 (19.8–23.3)	22.0 (20.0–23.0)	21.0 (19.5–22.5)	1.45	0.484
Height (cm)	178.5 (175.3–180.8)	177.0 (174.0–180.0)	179.0 (176.5–181.0)	0.89	0.641
Body mass (kg)	74.2 (70.1–78.2)	75.0 (71.3–78.7)	73.8 (70.4–77.3)	0.54	0.763
BMI (kg/m^2^)	23.3 (22.2–24.3)	23.9 (23.1–25.0)	23.0 (22.1–24.3)	1.12	0.571
Resting HR (bpm)	74.0 (71.0–77.0)	73.5 (70.0–76.5)	72.0 (69.5–75.5)	1.07	0.586
Physical activity (min/week)	62 (48–79)	65 (51–82)	58 (44–73)	1.18	0.554

Kruskal–Wallis *H* test. HR = heart rate; IPAQ = International Physical Activity Questionnaire; BMI = body mass index. No significant between-group differences were detected for any demographic variable (all *p* > 0.05).

### 2.4 Acute exercise protocols

All experimental sessions were conducted between 08:30 and 10:30 h to minimize circadian variation in hormonal and cognitive parameters (19). All exercise protocols and standardized drill sequences were directly administered and supervised by the investigator (CCA.) to ensure technical and procedural consistency across all sessions. Heart rate was continuously monitored using validated chest-strap telemetry (Polar H10, Polar Electro Oy, Kempele, Finland).

Basketball Protocol (High-Intensity Intermittent Exercise): Following a standardized 5-min dynamic warm-up (jogging, dynamic stretching, agility ladder drills), the BB group performed four 8-min bouts of structured basketball drills (fast-break sequences, two-on-two half-court plays, defensive transition drills, and full-court dribbling with jump stops), each separated by 2-min passive rest intervals, targeting 75–90% HRmax. Total active exercise duration was 32 min.

Tennis Protocol (Moderate-Intensity Continuous Exercise): Following an identical 5-min warm-up, the TEN group performed 35 continuous minutes of controlled court drills. Ball-feeding machines delivered forehand, backhand, and overhead balls at a tempo standardized to maintain 60–75% HRmax, with all lateral displacements, court coverage, and stroke mechanics supervised by a certified tennis coach. Total exercise duration was 35 min.

Control Protocol: Participants in the CON group remained seated in a quiet, climate-controlled room (22–24 °C) for 45 min, avoiding excessive movement, reading, or digital device use. Heart rate remained at resting levels throughout.

### 2.5 Blood sampling and ELISA analysis

Venous blood samples (5 mL each) were collected from the antecubital vein by a certified nurse at two time points: T1 (pre-exercise, 10 min before session onset) and T2 (post-exercise, within 5 min of session completion). Blood was drawn into serum-separating vacuum tubes (BD Vacutainer SST, Becton Dickinson, NJ, USA), allowed to clot at room temperature for 30 min, then centrifuged at 3,000 rpm for 15 min at +4 °C. Serum supernatants were aliquoted and stored at −80 °C until batch analysis.

Serum, rather than plasma, was selected as the analytical matrix for BDNF (as well as irisin and NRF2) for two reasons. First, serum is the most widely used matrix in the acute-exercise BDNF literature, allowing direct comparability of our findings with prior sport-specific studies. Second, approximately 99% of peripheral BDNF is stored within platelets and is released during clot formation; serum BDNF concentrations are consequently substantially higher than, and reflect a biologically distinct pool from, plasma BDNF, which better approximates free-circulating BDNF (20). Because platelet activation and release are themselves influenced by exercise intensity, serum BDNF may partly reflect exercise-induced platelet degranulation in addition to any centrally-relevant free BDNF fraction; this is acknowledged as an interpretive limitation in Section 4.

Serum irisin concentrations were measured using a commercially available human irisin/FNDC5 ELISA kit (Phoenix Pharmaceuticals, Inc., EIA-FNDC5-1, Burlingame, CA, USA). Serum BDNF concentrations were quantified using a sandwich ELISA kit (Boster Biological Technology, EK0309, Pleasanton, CA, USA). Serum NRF2 protein levels were determined using a solid-phase ELISA kit (Cloud-Clone Corp., SEG557Hu, Houston, TX, USA). All samples were assayed in duplicate. Mean intra-assay coefficients of variation (CV) were 4.2% for irisin, 5.1% for BDNF, and 5.8% for NRF2.

### 2.6 Cognitive performance assessment

Cognitive assessments were administered by a trained evaluator blinded to group assignment at T1 (15 min before exercise) and T2 (30 min after exercise completion). Two standardized instruments were used: (1) the Digit Span subtest from the WAIS-IV (21), assessing verbal working memory (maximum 30 points); and (2) the Stroop Color-Word Interference Test (22), assessing executive inhibitory control via mean incongruent-condition reaction time (ms), with alternate forms counterbalanced across T1 and T2 to control for practice effects.

### 2.7 Statistical analysis

All statistical analyses were performed using IBM SPSS Statistics Version 26.0. Shapiro–Wilk testing confirmed significant departures from normality across all outcome variables and group-time combinations (W range: 0.819–0.911; all *p* < 0.05); non-parametric methods were therefore used as the primary analytic approach. Within-group pre-to-post differences were evaluated using the Wilcoxon Signed-Rank test (effect size *r* = *Z*/√*N*). Between-group comparisons of exercise-induced difference scores (Δ = post − pre) were analyzed using the Kruskal–Wallis H test (effect size *ε*^2^), followed by pairwise Dunn-Bonferroni post-hoc tests (α_adjusted = 0.05/3 = 0.017).

As a supplementary robustness check addressing the reliance on difference scores, a linear mixed-effects model (Group × Time interaction, random intercept per subject, restricted maximum likelihood estimation) was fitted to the subject-level serum irisin dataset. This model corroborated the non-parametric findings: relative to Control, the Group × Time interaction was significant for both Basketball (*b* = 6.18, SE = 0.31, *z* = 19.90, *p* < 0.001) and Tennis (*b* = 4.44, SE = 0.31, *z* = 14.31, *p* < 0.001), indicating that the reliance on non-parametric difference-score analysis did not materially alter the study’s substantive conclusions for this outcome. Equivalent mixed-model analyses could not be performed for BDNF, NRF2, Digit Span, or Stroop RT within the scope of this revision because subject-level longitudinal data for these outcomes were not available to the corresponding authors at the time of resubmission; we recommend these analyses be completed on the full raw dataset as a further robustness step.

The *a priori* significance threshold for all analyses was set at *p* < 0.05 (two-tailed).

## 3 Results

### 3.1 Exercise session intensity verification

Mean heart rate during exercise sessions differed significantly across groups [H(2) = 52.14, *p* < 0.001]: BB: 162 bpm (IQR: 156–168 bpm); TEN: 147 bpm (IQR: 141–153 bpm); CON: 71 bpm (IQR: 68–74 bpm, resting). Using the traditional age-predicted maximum heart rate formula (220 − age) and the group median ages reported in Table 1, relative intensities were: BB 81.6% HRmax, TEN 74.2% HRmax, CON 35.7% HRmax. As requested during peer review, we additionally recomputed these values using the more contemporary Tanaka formula [HRmax = 208–0.7 × age; (23)]: BB 84.0% HRmax, TEN 76.3% HRmax, CON 36.7% HRmax. Both formulas confirm the intended intensity differential across all three conditions, including the Control group remaining at resting/very-light intensity. Pairwise post-hoc tests confirmed significant differences between all group pairs (all *p* < 0.001; Table 2).

**Table 2:** Exercise session heart rate and relative intensity by group.

Group	Session HR (bpm)	%HRmax (220 − age)	%HRmax (Tanaka, 208–0.7 × age)
Basketball	162 (156–168)	81.6%	84.0%
Tennis	147 (141–153)	74.2%	76.3%
Control	71 (68–74)	35.7%	36.7%

### 3.2 Within-group changes in serum irisin

The BB group exhibited a significant post-exercise increase in serum irisin [pre: 15.02 (IQR: 14.48–16.09) ng/mL vs. post: 21.31 (IQR: 20.39–22.85) ng/mL; *Z* = −3.92, *p* < 0.001, *r* = 0.62], a 41.9% elevation. The TEN group demonstrated a significant but smaller increase [pre: 15.23 (IQR: 14.60–16.37) ng/mL vs. post: 19.88 (IQR: 19.14–20.81) ng/mL; *Z* = −3.92, *p* < 0.001, *r* = 0.62], a 30.5% rise. No significant change was detected in the CON group [pre: 14.45 (IQR: 13.95–15.86) ng/mL vs. post: 15.00 (IQR: 14.12–15.98) ng/mL; *Z* = −0.48, *p* = 0.631, *r* = 0.11]. A supplementary linear mixed-effects model corroborated these findings, with significant Group × Time interactions for both Basketball (*b* = 6.18, *p* < 0.001) and Tennis (*b* = 4.44, *p* < 0.001) relative to Control (see Section 2.7; Table 3).

**Table 3:** Within-group serum irisin levels before and after acute exercise sessions (data: median [IQR]).

Group	Pre (ng/mL)	Post (ng/mL)	Δ (ng/mL)	Δ%	*Z*	*p*	*r*
Basketball	15.02 (14.48–16.09)	21.31 (20.39–22.85)	6.02 (5.20–6.88)	+41.9%	−3.92	<0.001***	0.62
Tennis	15.23 (14.60–16.37)	19.88 (19.14–20.81)	4.44 (3.90–4.97)	+30.5%	−3.92	<0.001***	0.62
Control	14.45 (13.95–15.86)	15.00 (14.12–15.98)	0.23 (−0.25–0.60)	+3.8%	−0.48	0.631	0.11

Wilcoxon Signed-Rank Test. *r* = *Z*/√*N* (effect size). Δ = Post − Pre. ***p < 0.001.

### 3.3 Within-group changes in serum BDNF

A significant and large-magnitude increase in BDNF was observed in the BB group [pre: 2,105.0 (IQR: 340.0) pg/mL vs. post: 3,450.0 (IQR: 520.0) pg/mL; *Z* = −3.92, *p* < 0.001, *r* = 0.62], a 63.9% elevation. The TEN group showed a significant but smaller increase [pre: 2,140.0 (IQR: 295.0) pg/mL vs. post: 2,780.0 (IQR: 410.0) pg/mL; *Z* = −3.92, *p* < 0.001, *r* = 0.62; +29.9%]. The CON group showed no significant BDNF change [pre: 2,090.0 (IQR: 310.0) pg/mL vs. post: 2,100.0 (IQR: 305.0) pg/mL; *Z* = −0.24, *p* = 0.810, *r* = 0.05; Table 4].

**Table 4:** Within-group serum BDNF concentrations before and after exercise (data: median [IQR]).

Group	Pre (pg/mL)	Post (pg/mL)	Δ (pg/mL)	Δ%	*Z*	*p*	*r*
Basketball	2,105.0 (340.0)	3,450.0 (520.0)	1,345.0 (215.0)	+63.9%	−3.92	<0.001***	0.62
Tennis	2,140.0 (295.0)	2,780.0 (410.0)	640.0 (150.0)	+29.9%	−3.92	<0.001***	0.62
Control	2,090.0 (310.0)	2,100.0 (305.0)	10.0 (45.0)	+0.5%	−0.24	0.810	0.05

BDNF = Brain-Derived Neurotrophic Factor. Wilcoxon Signed-Rank Test. *r* = *Z*/√*N*. ***p < 0.001.

### 3.4 Within-group changes in serum NRF2

Significant post-exercise NRF2 elevations were detected in BB [pre: 2.83 (IQR: 2.31–3.42) ng/mL vs. post: 4.91 (IQR: 4.28–5.63) ng/mL; *Z* = −3.92, *p* < 0.001, *r* = 0.62; +73.5%] and TEN [pre: 2.61 (IQR: 2.14–3.18) ng/mL vs. post: 3.84 (IQR: 3.31–4.52) ng/mL; *Z* = −3.87, *p* < 0.001, *r* = 0.61; +47.1%]. No significant change was observed in CON [pre: 1.94 (IQR: 1.52–2.38) ng/mL vs. post: 2.01 (IQR: 1.58–2.46) ng/mL; *Z* = −0.79, *p* = 0.428, *r* = 0.12; Table 5].

**Table 5:** Within-group serum NRF2 levels before and after exercise (data: median [IQR]).

Group	Pre (ng/mL)	Post (ng/mL)	Δ (ng/mL)	Δ%	*Z*	*p*	*r*
Basketball	2.83 (2.31–3.42)	4.91 (4.28–5.63)	2.08 (1.97–2.21)	+73.5%	−3.92	<0.001***	0.62
Tennis	2.61 (2.14–3.18)	3.84 (3.31–4.52)	1.23 (1.17–1.34)	+47.1%	−3.87	<0.001***	0.61
Control	1.94 (1.52–2.38)	2.01 (1.58–2.46)	0.07 (0.06–0.08)	+3.6%	−0.79	0.428	0.12

NRF2 = Nuclear Factor Erythroid 2-Related Factor 2. Wilcoxon Signed-Rank Test. *r* = *Z*/√*N*. ***p < 0.001.

### 3.5 Within-group changes in cognitive performance

Significant post-exercise improvements were observed in Digit Span total scores and Stroop RT in both the BB and TEN groups (all *p* < 0.001 and *p* < 0.01, respectively). The BB group exhibited larger cognitive gains: Digit Span improved from 14.0 (IQR: 12.0–16.0) to 17.5 (IQR: 15.5–19.5) points (*Z* = −3.82, *p* < 0.001, *r* = 0.60), and Stroop incongruent RT decreased from 682 (IQR: 624–740) ms to 597 (IQR: 551–643) ms (*Z* = −3.74, *p* < 0.001, *r* = 0.59). In the TEN group, Digit Span improved from 13.5 (IQR: 11.5–15.5) to 15.5 (IQR: 13.5–17.5) points (*Z* = −2.86, *p* = 0.004, *r* = 0.45), and Stroop RT declined from 695 (IQR: 640–752) ms to 641 (IQR: 591–688) ms (*Z* = −2.44, *p* = 0.015, *r* = 0.39). No significant cognitive changes were detected in the CON group for either Digit Span (*Z* = −0.50, *p* = 0.617) or Stroop RT (*Z* = −0.37, *p* = 0.714; Table 6).

**Table 6:** Cognitive performance outcomes before and after exercise (data: median [IQR]).

Test	Group	Pre	Post	*Z*	*p*	*r*
Digit Span (pts)	Basketball	14.0 (12.0–16.0)	17.5 (15.5–19.5)	−3.82	<0.001***	0.60
Digit Span (pts)	Tennis	13.5 (11.5–15.5)	15.5 (13.5–17.5)	−2.86	0.004**	0.45
Digit Span (pts)	Control	13.0 (11.0–15.0)	13.0 (11.5–15.5)	−0.50	0.617	0.08
Stroop RT (ms)	Basketball	682 (624–740)	597 (551–643)	−3.74	<0.001***	0.59
Stroop RT (ms)	Tennis	695 (640–752)	641 (591–688)	−2.44	0.015*	0.39
Stroop RT (ms)	Control	701 (643–757)	697 (640–754)	−0.37	0.714	0.06

Digit Span maximum score = 30 (combined forward + backward). RT = reaction time (lower = better). Wilcoxon Signed-Rank Test. *r* = *Z*/√*N*. *p < 0.05; **p < 0.01; ***p < 0.001.

### 3.6 Between-group comparisons of difference scores

Significant between-group differences were detected for all five outcome variables. Post-hoc Dunn-Bonferroni pairwise comparisons consistently confirmed the hierarchical ordering of BB > TEN > CON for all biomarker and cognitive difference scores (all *p* < 0.01 for BB vs. TEN; all *p* < 0.001 for exercise groups vs. CON). Large effect sizes (*ε*^2^) were obtained for all biomarkers and moderate-to-large effect sizes for cognitive outcomes (Table 7).

**Table 7:** Between-group comparison of exercise-induced difference scores (Δ = Post − Pre). Kruskal–Wallis *H* test with post-hoc Dunn-Bonferroni.

Outcome	BB Δ (IQR)	TEN Δ (IQR)	CON Δ (IQR)	*χ*^2^(H)	*p*	*ε* ^2^	BB vs TEN	BB vs CON	TEN vs CON
Δ Irisin (ng/mL)	6.02 (1.68)	4.44 (1.07)	0.23 (0.60)	47.38	<0.001***	0.79	*p* < 0.01**	*p* < 0.001***	*p* < 0.001***
Δ BDNF (pg/mL)	1,345.0 (215.0)	640.0 (150.0)	10.0 (45.0)	49.12	<0.001***	0.82	*p* < 0.01**	*p* < 0.001***	*p* < 0.001***
Δ NRF2 (ng/mL)	2.08 (0.24)	1.23 (0.17)	0.07 (0.02)	44.67	<0.001***	0.75	*p* < 0.01**	*p* < 0.001***	*p* < 0.001***
Δ Digit Span (pts)	3.5 (2.0)	2.0 (1.5)	0.0 (0.5)	38.92	<0.001***	0.65	*p* < 0.01**	*p* < 0.001***	*p* < 0.001***
Δ Stroop RT (ms)	−85 (28)	−54 (22)	−4 (11)	35.48	<0.001***	0.59	*p* < 0.01**	*p* < 0.001***	*p* < 0.001***

BB = Basketball; TEN = Tennis; CON = Control. *ε*^2^ = epsilon-squared. Bonferroni-adjusted *α* = 0.017. Positive Δ = increase; negative Δ Stroop RT = improvement. **p < 0.01; ***p < 0.001.

A correlation analysis between biomarker changes (ΔIrisin, ΔBDNF, ΔNRF2) and cognitive-performance changes (ΔDigit Span, ΔStroop RT), and a within-group dose–response analysis relating individual exercise-session heart-rate intensity to individual biomarker and cognitive changes, were not performed in the present study, as subject-level continuous heart-rate telemetry and the full subject-level dataset needed for a robust multi-variable correlation matrix were not retained in analyzable form. We report this explicitly rather than inferring an association that was not statistically tested, and we identify it as a priority direction for future studies with prospectively archived subject-level data (see Section 4).

## 4 Discussion

This study provides comparative evidence that a single acute session of high-intensity intermittent basketball exercise elicits significantly greater elevations in serum irisin, BDNF, and NRF2, and produces superior improvements in working memory capacity and executive inhibitory control, compared to a moderate-intensity continuous tennis session in sedentary young adults. The sedentary control group exhibited no significant changes in any biomarker or cognitive measure, indicating that observed effects were attributable to the exercise stimulus rather than circadian fluctuation, familiarization, or practice effects. A consistent hierarchical ordering of Basketball > Tennis > Control was observed across all five outcome variables, with large effect sizes, providing quantitative support for an intensity-dependent model of exercise-induced neurotrophic and antioxidant activation. However, because basketball and tennis differ not only in metabolic intensity but also in motor-skill demands and open- versus closed-skill cognitive load, the present between-groups design cannot fully isolate the metabolic contribution from these other sources of difference (see Limitations).

The irisin response observed in the BB group (+41.9%) exceeds that of the TEN group (+30.5%), consistent with the established role of exercise intensity and type II myofiber recruitment in driving PGC-1α-mediated FNDC5 upregulation and irisin secretion (3). Basketball’s intermittent demands necessitate repeated maximal-intensity efforts recruiting high-threshold motor units and type IIx myofibers, which may plausibly explain the larger irisin response, though this mechanism was not directly measured in the present study and remains an inference rather than a demonstrated pathway here.

The substantially greater BDNF elevation in BB (+63.9%) versus TEN (+29.9%) is broadly consistent with prior meta-analytic findings (9, 24) that sport-specific exercise intensity may modulate the magnitude of peripheral BDNF release. Several non-mutually-exclusive mechanisms could plausibly underlie this differential: higher blood lactate concentrations during high-intensity intermittent exercise may facilitate lactate-mediated BDNF transcription via astrocytic SIRT1 signaling (25); catecholamine surges during maximal effort may stimulate *β*₂-adrenergic-receptor-mediated BDNF expression (26); and exercise-induced irisin may upregulate hippocampal BDNF via ERK1/2 signaling (6). We emphasize that lactate and catecholamine concentrations were not measured in the present study; these mechanisms are therefore presented as plausible, literature-based interpretations rather than findings directly demonstrated by our data.

The differential NRF2 response (BB: +73.5%; TEN: +47.1%) may plausibly reflect intensity-dependent ROS generation during muscular contraction and consequent KEAP1/NRF2 nuclear translocation (4, 27), consistent with the hormetic principle that appropriate oxidative stress can act as a beneficial adaptive trigger (28). As with the BDNF discussion above, direct markers of oxidative stress (e.g., ROS, KEAP1 oxidation) were not measured, and this interpretation should be read as a plausible mechanism rather than a demonstrated one.

The cognitive improvements particularly the larger working memory (Digit Span) and inhibitory control (Stroop RT) gains in the BB group are temporally consistent with the larger neurotrophic and antioxidant changes observed in this group, and may plausibly relate to BDNF-mediated synaptic modulation of hippocampal and prefrontal circuitry (2, 11, 29). However, we did not perform a formal statistical test (e.g., correlation or mediation analysis) linking individual biomarker changes to individual cognitive changes in the present dataset (see Section 3.6), and this association should therefore be understood as a literature-consistent hypothesis rather than a demonstrated within-study relationship. Because peripheral (serum) BDNF is an indirect proxy for central neurotrophic activity, and because serum BDNF partly reflects platelet-releasable rather than purely free-circulating BDNF (20), these cognitive-biomarker interpretations should be treated with appropriate caution.

An additional, non-mutually-exclusive explanation for the larger cognitive gains in the basketball condition is that basketball imposes a substantially greater perceptual-cognitive load than tennis as practiced in this protocol: basketball’s open-skill, reactive, rapidly changing tactical demands (fast breaks, defensive transitions) require continuous decision-making, whereas the tennis protocol used ball-feeding machines to standardize a more predictable, closed-skill stroke pattern. This greater cognitive engagement during the basketball sessions could independently contribute to superior post-exercise cognitive performance, separate from any biomarker-mediated mechanism, and is noted here as an important limitation of the between-sport comparison.

Several methodological strengths distinguish this study. The operational definition of sedentary status via IPAQ-SF and a six-month inactivity criterion ensures genuine baseline physiological deconditioning across all groups. The use of validated ELISA kits with documented intra-assay CVs below 6%, combined with duplicate analysis and batch processing, minimizes analytical variance. The application of G*Power-validated sample sizes, non-parametric statistics appropriate to the confirmed non-normal distributions, and a supplementary mixed-effects model for serum irisin, strengthens confidence in the primary biomarker findings.

## 5 Limitations

This study has several limitations. First, the exclusive enrollment of young adult males precludes generalization to female or older populations, and all conclusions should be restricted to sedentary young adult males aged 18–25 years similar to the present sample; broader population-level claims are not supported by this design. Second, basketball- and tennis-specific motor familiarity accumulated through informal, non-competitive exposure was not formally quantified, and residual between-sport differences in movement efficiency and skill cannot be excluded as a contributor to the observed biomarker and cognitive differences, alongside metabolic intensity. Third, the parallel-groups (non-crossover) design means that between-group differences may reflect a combination of the sport-specific exercise paradigm and between-subject individual variability; a crossover or repeated-exposure design would be needed to more fully isolate these sources. Fourth, we did not assess post-exercise plasma volume changes (hemoconcentration); acute exercise can transiently reduce plasma volume via fluid shifts and sweating, which may have inflated the apparent magnitude of serum biomarker increases independent of true secretion, particularly in the higher-intensity basketball condition, and future studies should apply a plasma-volume correction (e.g., the Dill & Costill method). Fifth, serum BDNF reflects a platelet-releasable pool that may not fully track free-circulating or centrally-relevant BDNF (20), and this should be considered when interpreting the magnitude and meaning of the reported BDNF changes. Sixth, as noted in Section 3.6, biomarker–cognition and intensity–response correlation analyses were not performed due to data availability constraints and are recommended as a priority for future supplementary analysis. Seventh, basketball’s greater open-skill cognitive load relative to the tennis protocol used here may have contributed to the larger cognitive gains independent of biomarker-mediated mechanisms. Eighth, a fully documented, prospectively archived randomization and allocation-concealment protocol, along with a complete CONSORT-style participant flow record, was not retained for the present cohort; while group allocation preceded baseline testing, we cannot reconstruct the precise sequence-generation and concealment procedure for this revision, and this is acknowledged as a methodological limitation. Ninth, psychiatric-diagnosis exclusion relied on participant self-report during the intake interview rather than a standardized structured clinical instrument, which may have permitted under-reporting. Finally, the acute design does not permit conclusions regarding chronic training adaptations, dose–response kinetics, or durability of cognitive effects, and comprehensive dietary/nutrient profiling was not performed. We have adopted prospective trial registration, formal randomization documentation, and structured psychiatric screening as standard practice in our ongoing and future work to address these gaps.

## 6 Conclusion

In sedentary young adult males aged 18–25 years, a single acute session of high-intensity intermittent basketball exercise produced significantly greater elevations in serum irisin, BDNF, and NRF2 concentrations, and superior improvements in working memory capacity and executive inhibitory control, compared to a moderate-intensity continuous tennis protocol. The hierarchical response pattern of Basketball > Tennis > Control, observed across all five primary outcomes, is consistent with an intensity-dependent model of muscle-brain axis activation, although sport-specific motor and cognitive-load factors may also contribute and could not be fully disentangled in the present design. These findings should be interpreted as applicable to populations resembling the present sample rather than generalized broadly, and future research using crossover designs, plasma-volume correction, and formal biomarker–cognition correlation analyses is recommended to clarify the mechanisms underlying these observations.

## Data Availability

The original contributions presented in the study are included in the article/supplementary material, further inquiries can be directed to the corresponding author.
